# Correlation Between Foot Posture and Hamstring Muscle Tightness

**DOI:** 10.7759/cureus.42046

**Published:** 2023-07-17

**Authors:** Purva Gulrandhe, Vaishnavi Yadav, Waqar M Naqvi

**Affiliations:** 1 Department of Physiotherapy, Ravi Nair Physiotherapy College, Datta Meghe Institute of Medical Sciences, Wardha, IND; 2 Department of Physiotherapy, Datta Meghe Institute of Medical Sciences, Wardha, IND; 3 Department of Physiotherapy, College of Health Sciences, Gulf Medical University, Ajman, ARE

**Keywords:** foot posture, biomechanics, foot posture index, muscle tightness, hamstring

## Abstract

Background

The hamstring muscle is related to the lumbar spine, pelvic, and lower limb movement dysfunction, as well as low back pain and abnormal gait. The kinematic chain's distal elements dysfunctions may affect the body's proximal segments. There is a biomechanical connection between the foot and proximal segments of the body and its effect on the body's functional status, but there is a lack of research that focuses on the correlation between foot posture and hamstring muscle tightness. The study aimed to find the correlation between hamstring muscle tightness and foot posture using the foot posture index (FPI).

Methods

After obtaining ethical committee approval, necessary authorization was obtained from relevant authorities to proceed with participant screening. Informed consent was obtained from every participant, accompanied by a comprehensive explanation of the study. Screening of participants was conducted based on specific inclusion and exclusion criteria. These criteria were crucial for selecting a homogeneous sample and ensuring the study's objectives were met. The assessment of foot posture was carried out using FPI, and hamstring tightness was examined using an active knee extension test.

Result and discussion

In our study, which included 188 participants aged between 18 and 25 (mean age: 21.91±1.97), we examined the correlation between FPI and active knee extension (AKE) test results. Using Pearson's correlation coefficient, we found a statistically significant correlation between the FPI and AKE test results. For the right side, the r-value was 0.678 (p-value = 0.0001); for the left side, the r-value was 0.653 (p-value = 0.0001); and for the total, the r-value was 0.663 (p-value = 0.0001). These findings indicate a significant relationship between the FPI and AKE test results.

Conclusion

The findings of our study revealed a significant relationship between hamstring tightness and pronation of the foot, as measured by the FPI. Understanding this relationship is crucial as it sheds light on the potential impact of hamstring tightness on foot biomechanics. By establishing this link, our study contributes to the body of knowledge surrounding the prevention of alterations in foot biomechanics. It highlights the importance of addressing hamstring tightness to mitigate potential foot pronation issues. Moreover, the study serves as a stepping stone for future research endeavors. It lays the groundwork for further large-scale investigations that encompass a broader range of age groups.

## Introduction

The hamstring muscles serve as extensors for the hip and flexors for the knee. During the swing phase of the gait cycle, they become engaged in the final 25% as hip extension initiates. Their activity persists for 50% of the swing phase, where they actively contribute to hip extension and counteract knee extension. It is important to note that achieving full hip flexion is only possible when the knee joint is flexed as well. This limitation is due to the relatively short length of the hamstring muscles. Hamstring tightness is relatively common among college students aged 18-25. Tight hamstrings are associated with dysfunction in the lumbar spine, pelvis, and lower limb, as well as conditions such as low back pain and abnormal gait [1]. Apart from causing a reduced range of motion, hamstring tightness can contribute to various orthopedic conditions, such as hamstring strain, plantar fasciitis, and low back pain. Muscle tightness affects the ability of the hamstring muscles to maintain a constant length-tension relationship and absorb stress [2]. This decreased flexibility creates a cycle of a limited range of motion and increased postural abnormalities. Muscle tension also constricts blood vessels, leading to suboptimal functioning [2]. Faulty motor control patterns are associated with hamstring tightness, resulting in suboptimal firing of postural muscles and hamstrings acting more as stabilizers than primary movers. This fundamental alteration in function leads to the presentation of hamstring tightness [2,3]. In research settings, passive straight leg raise tests and active knee extension (AKE) are commonly considered gold standards. However, clinical settings often utilize assessments such as the v-sit and reach tests and finger-to-floor distance to measure apparent hamstring flexibility and tightness [4,5]. Hamstring muscle complex (HMC) tightness can contribute to various issues, including muscle strains, loss of lumbar spine curvature, sacroiliac joint disorders, and plantar fasciitis. These problems primarily arise due to misalignment of the body and imbalanced force distribution between muscles and joints [6].

Foot posture, also referred to as foot type in the literature, and altered lower extremity mobility can also lead to injury. Researchers have predominantly focused on three methodologies - kinetics or plantar pressures, electromyography, and kinematics - to analyze the biomechanics of the lower limb in relation to foot position and injury [7]. Dysfunctions in the distal elements of the kinematic chain can impact the proximal segments of the body. It is a crucial component of motor control for optimal biomechanical function, energy transfer, and joint loading minimization in the musculoskeletal system. It also enables the maintenance of proper movement patterns. The relationship between the distal segments of the body and core muscles is well-established, as the core's stability influences mobility and energy transmission from proximal to distal segments [8,9]. Dysfunctions in the plantar region of the foot can manifest as issues in the upper aspects of the anatomy trains. Indications of such dysfunction include knee hyperextension, shortening, increased cervical lordosis, reduced hamstring muscle flexibility, and decreased lumbar lordosis [8]. Distal kinematic chain dysfunctions can affect proximal body segments, and foot pronation, for example, can alter the biomechanics of the upper joints [8]. The superficial backline, comprising the hamstring muscles and plantar short foot muscles, should be viewed from a multidisciplinary view, considering the interconnectedness of myofascia according to the anatomy trains paradigm [8]. Evidence of this interconnectedness is supported by the reported instantaneous increase in hamstring flexibility following a single self-myofascial-release technique using a tennis ball on the plantar portion of the foot. Weakness in proximal muscle stability can result in reduced movement pattern efficiency, compensatory patterns of movement, and tissue overloading [8,10,11]. While there is literature supporting the biomechanical connection between the foot and proximal segments of the body and its impact on functional status, research focusing on the correlation between foot posture and hamstring muscle tightness remains limited. The aim of this study was to investigate the relationship between hamstring muscle tightness and foot posture. The objectives of the study were to assess the degree of hamstring tightness in healthy individuals, to evaluate foot posture, and to determine the correlation between hamstring tightness and foot posture.

## Materials and methods

The study commenced after obtaining approval from the ethical committee (approval number - DMIMS(DU)/IEC/2022/916), ensuring that this research adhered to ethical standards and guidelines. This study utilized a cross-sectional correlational design. Subsequently, authorization was sought from the appropriate authorities to proceed with participant screening. Informed consent was obtained from each participant, where they were provided with a clear explanation of the study objectives, procedures, potential risks, and benefits, allowing them to make an informed decision regarding their participation. 

Participants who expressed their willingness to participate in the study underwent a comprehensive screening process. This screening involved evaluating potential participants based on predetermined inclusion and exclusion criteria. Inclusion criteria included individuals with hamstring tightness, healthy individuals aged 18-25, and both males and females. Exclusion criteria encompassed recent surgery, fractures within the past six months, lower leg discrepancy (> 1 or 1.5 cm), and a history of neurological and musculoskeletal problems. These criteria ensured the selection of participants with relevant characteristics for studying the relationship between hamstring tightness and foot posture while minimizing confounding factors. Hamstring tightness was assessed by actively extending the knee. To assess hamstring tightness, the AKE test was performed, and knee movement ranges were determined using a standard, double-arm, full-circle transparent plastic goniometer. The foot posture index (FPI) was used to measure foot posture by assessing specific parameters. Rearfoot parameters, including the talar head position, malleolar curvature, calcaneal angle, talonavicular coverage, and forefoot-to-rearfoot alignment, were evaluated. Forefoot parameters, such as talo-navicular congruence, medial arch height, and forefoot abduction/adduction, were also measured. Each parameter was visually examined, and a score ranging from -2 to +2 was assigned. These scores were then summed to determine the overall foot posture. The FPI scores provided insights into the alignment and biomechanics of the foot, representing varying degrees of supination, pronation, or neutrality. In this study, the FPI scores were analyzed in relation to measures of hamstring tightness to investigate any potential correlation between the two variables. By examining the data from both assessments, the study aimed to determine if a relationship existed between hamstring tightness and specific foot postures, shedding light on their potential interaction and influence.

Statistical analysis

Descriptive and inferential statistical analyses were conducted to analyze the data collected in the study. Pearson's correlation coefficient was utilized to examine the correlation between variables. The statistical software used for the analysis was SPSS 27.0. The level of significance was set at p < 0.05, indicating a statistically significant result.

## Results

We included 188 participants in our study between the age group 18-25. The mean age was 21.91 ± 1.97. Participants included were aged 18 years (15 participants, 8.0%), 19 years (11 participants, 5.9%), 20 years (16 participants, 8.5%), 21 years (33 participants, 17.6%), 22 years (28 participants, 14.9%), 23 years (47 participants, 25.0%), 24 years (19 participants, 10.1%), and 25 years (19 participants, 10.1%). Table 1 shows the age-wise (years) distribution of participants. 

**Table 1:** Age-wise (years) distribution of participants

Age Group (years)	No of participants	Percentage
18 years	15	8.0
19 years	11	5.9
20 years	16	8.5
21 years	33	17.6
22 years	28	14.9
23 years	47	25.0
24 years	19	10.1
25 years	19	10.1
Total	188	100.0
Mean±SD	21.91 ± 1.97 (18-25 years)	

In our study, male participants included were 79 with 42.02%, and female participants included were 109 with 57.98%. Table 2 represents the gender-wise distribution of participants. 

**Table 2:** Gender-wise distribution of participants

Gender	No of patients	Percentage
Male	79	42.02
Female	109	57.98
Total	188	100.0

**Figure 1:**
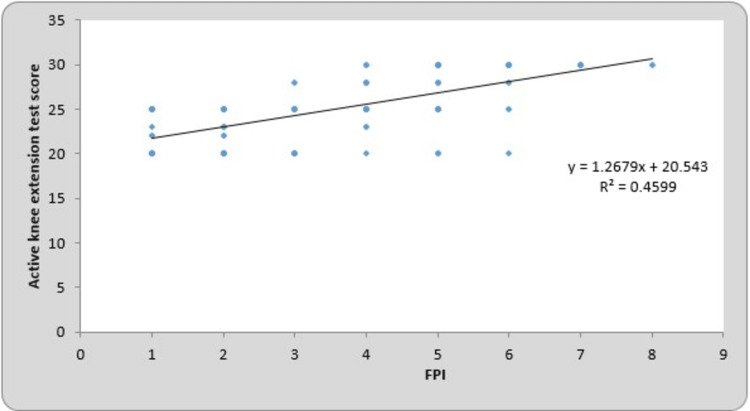
Correlation between the FPI and active knee extension test score of the right side

**Figure 2:**
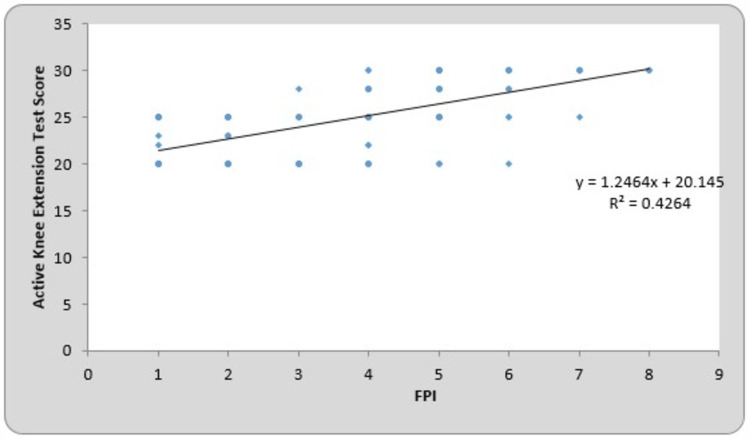
Correlation between the FPI and active knee extension test score of the left side

**Figure 3:**
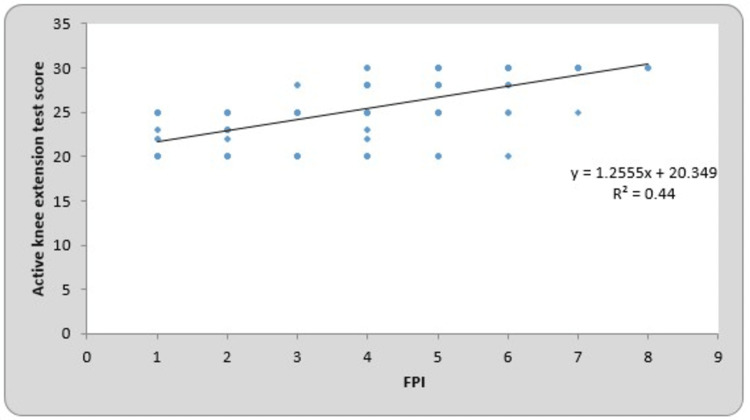
Correlation between the FPI and active knee extension test score in total participants

The mean of the FPI for the right and left sides were 3.23 ± 1.85 and 3.27 ± 1.89, respectively. The mean of the AKE test scores for the right and left sides were 24.64 ± 3.47 and 24.22 ± 3.61, respectively. By using Pearson’s correlation coefficient for the FPI and AKE test, the r-value for the right and left sides was 0.678 (p-value = 0.0001) and 0.653 (p-value = 0.0001), which is statistically significant. The total mean of the FPI was 3.25 ± 1.87, and the AKE test score was 24.43 ± 3.54. By using Pearson’s correlation coefficient for the FPI and AKE test, the r-value for the total mean was 0.663 (p-value = 0.0001), which is statistically significant. Table 3 presents the correlation between the FPI and AKE test scores on the right- and left-side Pearson’s correlation coefficient. The line charts showing the correlation between the FPI and AKE test scores of the right side, left side, and total participants are presented in Figures 1-3, respectively.

## Discussion

Although there is a plausible biomechanical and physiological rationale for the hypothesized connection between foot posture and hamstring muscle tightness, conclusive evidence regarding this relationship is lacking in large prospective studies. Therefore, the objective of our study was to investigate the correlation between hamstring muscle tightness and foot posture, utilizing the FPI and AKE test. A total of 188 participants were evaluated, including 109 females (42.02%) and 79 males (57.98%). The age distribution revealed a mean ± SD of 21.91 ± 1.97 years (18-25 years).

Sanchis-Sales et al. conducted a study examining the dynamic stiffness of foot joints during walking in the sagittal plane to better understand how static foot posture contributes to injury occurrence. They utilized FPI to assess the static foot postures of 70 healthy adult males. Their findings indicated that highly pronated feet exhibited a significantly reduced range of motion at the ankle and metatarsophalangeal joints compared to highly supinated feet, as well as a significantly increased range of moments. Static foot positions with greater dynamic stiffness during propulsion and higher absorbed work were associated with a doubled risk of injury [12]. Buldt et al. conducted a systematic study and found that individuals with pes planus (flat feet) displayed increased lower limb mobility during walking. Additionally, there is evidence linking higher frontal plane motion of the rearfoot to planus foot posture [7].

Kirmizi et al. conducted a study on 60 healthy individuals to assess the intra-rater and test-retest reliability of various clinical procedures for measuring foot posture. Among a group of 60, 30 individuals were randomly selected to assess interrater reliability. The study concluded that navicular drop showed greater reliability compared to other conventional procedures. Although the Foot Posture Index-6 demonstrated excellent intra-rater reliability, its interrater reliability was only moderate. These findings may provide physicians and researchers with a reliable method for implementing foot posture evaluation [13,14]. Buldt et al., in a systematic review of lower limb kinematics and foot posture during walking, identified seven studies demonstrating a relationship between foot kinematics and posture. They found evidence indicating that increasing planus foot posture was positively associated with greater frontal plane motion of the rearfoot. However, there was limited evidence linking increased lower limb movement and pes planus during walking, mainly due to study variability and modest effect sizes [7,15]. According to Kwon et al., excessive foot pronation not only contributes to musculoskeletal issues but also leads to elongation of the plantar fascia, posterior tibial muscle, and calcaneonavicular ligament while reducing the medial longitudinal arch and increasing internal rotation of the thigh. Furthermore, it can affect the length of the lower extremities. Such misalignment in the lower extremities can result in imbalances and increased pressure on the patellofemoral joint, leading to hamstring muscle shortening [16]. One limitation of our study was that we did not assess variables such as ankle extension and hallucis extension, which could have provided a more comprehensive understanding of the relationship between hamstring tightness and foot posture. Additionally, our study had a small sample size and a limited age range, reducing the generalizability of our findings. Future studies should consider including a larger and more diverse sample to increase the external validity of the results.

## Conclusions

In conclusion, our study provides compelling evidence supporting a significant relationship between hamstring tightness and pronation of the foot, as assessed through the FPI. This finding has important implications for preventing and addressing alterations in foot biomechanics. By incorporating regular hamstring stretches into everyday routines, individuals can potentially mitigate the negative effects of hamstring tightness on foot pronation, thus promoting optimal foot function and reducing the risk of related injuries.

Furthermore, the results of this study open up new avenues for future research in this field. By incorporating diverse age groups, researchers can gain a more comprehensive understanding of how these variables interact and whether the relationship holds true across various stages of life. Additionally, investigating the long-term effects of regular hamstring stretching and appropriate footwear on foot biomechanics would be beneficial. This could involve longitudinal studies that track individuals over an extended period to determine if consistent hamstring stretching and suitable footwear can effectively prevent or even reverse alterations in foot biomechanics associated with hamstring tightness.
